# Genome annotation for clinical genomic diagnostics: strengths and weaknesses

**DOI:** 10.1186/s13073-017-0441-1

**Published:** 2017-05-30

**Authors:** Charles A. Steward, Alasdair P. J. Parker, Berge A. Minassian, Sanjay M. Sisodiya, Adam Frankish, Jennifer Harrow

**Affiliations:** 1 Congenica Ltd, Wellcome Genome Campus, Hinxton, Cambridge, CB10 1DR UK; 2 The Wellcome Trust Sanger Institute, Wellcome Genome Campus, Hinxton, Cambridge, CB10 1SA UK; 3 Addenbrooke’s Hospital and University of Cambridge, Cambridge, CB2 0QQ UK; 4 Department of Pediatrics (Neurology), University of Texas Southwestern, Dallas, TX USA; 5 Program in Genetics and Genome Biology and Department of Paediatrics (Neurology), The Hospital for Sick Children and University of Toronto, Toronto, Canada; 6 Department of Clinical and Experimental Epilepsy, UCL Institute of Neurology, London, WC1N 3BG UK; 7 Chalfont Centre for Epilepsy, Chesham Lane, Chalfont St Peter, Buckinghamshire, SL9 0RJ UK; 8 European Molecular Biology Laboratory, European Bioinformatics Institute, Wellcome Genome Campus, Hinxton, Cambridge, CB10 1SD UK; 9 Illumina Inc, Great Chesterford, Essex, CB10 1XL UK

## Abstract

The Human Genome Project and advances in DNA sequencing technologies have revolutionized the identification of genetic disorders through the use of clinical exome sequencing. However, in a considerable number of patients, the genetic basis remains unclear. As clinicians begin to consider whole-genome sequencing, an understanding of the processes and tools involved and the factors to consider in the annotation of the structure and function of genomic elements that might influence variant identification is crucial. Here, we discuss and illustrate the strengths and weaknesses of approaches for the annotation and classification of important elements of protein-coding genes, other genomic elements such as pseudogenes and the non-coding genome, comparative-genomic approaches for inferring gene function, and new technologies for aiding genome annotation, as a practical guide for clinicians when considering pathogenic sequence variation. Complete and accurate annotation of structure and function of genome features has the potential to reduce both false-negative (from missing annotation) and false-positive (from incorrect annotation) errors in causal variant identification in exome and genome sequences. Re-analysis of unsolved cases will be necessary as newer technology improves genome annotation, potentially improving the rate of diagnosis.

## Background

Advances in genomic technologies over the past 20 years have provided researchers with unprecedented data relating to genome variation in different diseases [1]. However, even after whole-exome sequencing (WES), the genetic basis for a particular phenotype remains unclear in a considerable proportion of patients. Here, we examine how genomic annotation might influence variant identification, using examples mostly from both common and rarer neurological disorders. We highlight why the present technology can fail to identify the pathogenic basis of a patient’s disorder, or produce an incorrect result where the wrong variant is labelled as causative. For these reasons, we believe it is important to re-analyse unresolved cases as newer technology and software improve gene and genome annotation. The aim of this paper is to make common genomic techniques accessible to clinicians through the use of figures and examples that help to explain genome sequencing, gene classification and genome annotation in the context of pathogenic sequence variation. Finally, we discuss how new genomic techniques will improve our ability to identify pathogenic sequence variation.

## Genome sequencing

The Human Genome Project (HGP) was launched officially in 1987 by the US Department of Energy to sequence the approximately 3 billion base-pairs (bp) that constitute the human genome [2]. The first draft sequence was published in 2001 and computational annotation, a process that attributes a biological function to the genomic elements, described 30,000 to 40,000 protein-coding genes across 22 pairs of autosomes and the X and Y sex chromosomes in a genome of 2.9 billion bases (gigabases, Gb) [2]. The precise size and gene count of the reference human genome remains uncertain to this day because sequence gaps remain, while the classification of genes becomes more refined [3]. Consequently, additions are continually made to the genome to fill sequence gaps [4]. The most recent published estimates suggest that just under 20,000 protein-coding genes [5] are present in a genome of approximately 3.1 Gb [6]. The HGP enabled initial research examining sequence variation on chromosome 22 [7], to more recent medical advances that now see DNA sequencing used routinely in large-scale research programs, such as the Deciphering Developmental Disorders (DDD) study [8, 9]. Sequencing for the HGP used the chain terminator method [10], more commonly known as ‘Sanger sequencing’, and owing to the better-quality sequence data and read-length associated with Sanger sequencing compared with current sequencing technologies, Sanger sequencing is still used to confirm sequence variants [11].

Current methods for producing the raw sequence data for whole-genome sequencing (WGS) are placed into two categories based upon the length of the nucleotide sequence produced, or sequence ‘read’. Short-read technology comes from Illumina Inc. [12] and uses well-established chemistry to identify the sequence of nucleotides in a given short segment of DNA. Illumina sequencing platforms such as the HiSeq X produce base-pair reads of lengths from 150 to 250 bp in a given DNA segment and are used to read sequences from both ends of a DNA fragment. This ‘next-generation’ technology is a dramatic improvement over older Sanger sequencing methods that produced longer reads but at much higher cost [13]. More recently, ‘third-generation’ technologies from Pacific Biosciences (PacBio) and Oxford Nanopore are gaining users and making an impact. These third-generation methods generate longer reads, up to tens of thousands of base-pairs per read, but with higher error rates.

The speed of DNA sequencing, the amount of sequence that can be produced and the number of genomes that can be sequenced have increased massively with next-generation sequencing (NGS) techniques [14]. Such advances have enabled large collaborative projects that look at variation in a population, such as the 1000 Genomes Project [15], as well as those investigating the medical value of WGS, such as the UK 100,000 Genomes Project [16]. It is hoped that WGS will facilitate the research, diagnosis and treatment of many diseases.

**Fig. 1:**
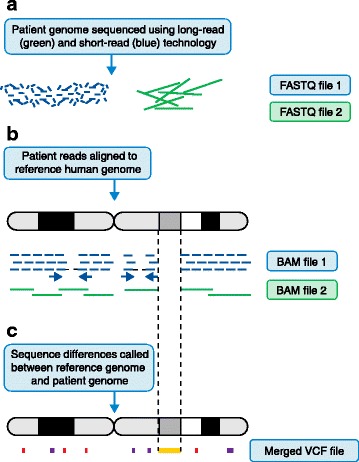
The genome analysis pipeline. Note that, for clarity, some steps have been omitted. Figure illustrations are not to scale and are only meant to be illustrative of the differences between short- and long-read sequencing. **a** Unaligned reads from sequencing machines are stored as FASTQ file formats. This is a text-based format for storing both a DNA sequence and its corresponding quality scores. **b** Reads are aligned to the genome. Short reads provide deep coverage, whereas reads that have been sequenced from both ends (*blue arrows*) help to orientate unaligned contigs. It is difficult to align short reads confidently across repetitive sequences when the repeating genome sequence is longer than the sequence read. Long-read sequences help to order contigs across larger regions, particularly with repetitive sequences, but do not provide the necessary depth needed to be confident of calling a base at a certain position. Note that there is a large region where there is no read coverage at all. This is indicative of structural variation. Here, the patient has a large deletion with respect to the reference genome. Once the reads have been aligned to the reference genome they are stored in a BAM file. A BAM file (.bam) is the binary version of a sequence alignment map (SAM file format). The latter is a tab-delimited text-based format for storing DNA sequences aligned to a reference sequence. **c** The Variant Call Format (VCF) specifies the format of a text file used in bioinformatics for storing genetic sequence variations. VCF files are much smaller than FASTQ and BAM files. Note that single-nucleotide variants (SNVs) and small insertions and deletions (‘indels’) are illustrated as *red* and *purple blocks*, whereas a much larger structural variant is indicated by an *orange block*

Once a patient genome has been sequenced, it needs to be aligned to the reference genome and analysed for variants. Typically, software algorithms such as the Burrows-Wheeler Aligner (BWA) are used for short- [17] and long-read [18] alignment and the Genome Analysis Toolkit (GATK) is used to identify or ‘call’ sequence variants [19]. Figure 1 illustrates a typical genome analysis pipeline, describing the different file formats commonly used—FASTQ [20], BAM [21] and VCF [22].

Pathogenic sequence variation can range in size from single-nucleotide variants (SNVs), small insertions and deletions (‘indels’) of fewer than 50 base-pairs in length, to larger structural variants (SVs) [23], which are generally classified as regions of genomic variation greater than 1 kb, such as copy-number variants (CNVs), insertions, retrotransposon elements, inversions, segmental duplications, and other such genomic rearrangements [24, 25]. Currently, the consequence of non-synonymous variants of the protein-coding elements only can be routinely automatically predicted by algorithms such as SIFT and PolyPhen [26], yet many different types of variants are implicated in disease. As sequencing techniques begin to move away from ‘gene panel’ testing to WGS, it is crucial to understand the structure of genes and any regulatory features that might lie within intra/intergenic regions as changes in any of these regions might have a crucial impact on the function of a gene.

Recently, the American College of Medical Genetics and Genomics (ACMG) recommended a set of standards and guidelines to help medical geneticists assign pathogenicity using standardized nomenclature and evidence used to support the assignment for Mendelian disorders [27]. For example, the terms ‘mutation’ and ‘polymorphism’ have often been used misleadingly, with assumptions made that ‘mutation’ is pathogenic, whereas ‘polymorphism’ is benign. As such, one recommendation that ACMG makes is that both these terms are replaced by ‘variant’, with the following modifiers (1) pathogenic, (2) likely pathogenic, (3) uncertain significance, (4) likely benign, or (5) benign [27]. As such, here, we use the term variant. A standard gene-variant nomenclature is maintained and versioned by the Human Genome Variation Society (HGVS) [28]. Both ACMG and HGVS examples are illustrated in Table 1.

**Table 1:** Examples of disease-causing variation with associated HGVS nomenclature

Location	Gene	Variation	HGVS nomenclature	ACMG clinical significance	Associated disorder	Reference
5′ UTR	*FMR1*	Expansion	NM_002024.5(FMR1):c.-128_-126(200)	Pathogenic	Fragile X syndrome	[186]
CDS	*GRIN2A*	Nonsense	NM_000833.4(GRIN2A):c.2041C > T (p.Arg681Ter)	Pathogenic	Idiopathic focal epilepsy (IFE) with rolandic spikes is the most common childhood epilepsy	[187]
CDS	*GABRB3*	Missense	NM_021912.4(GABRB3):c.745C > A (p.Gln249Lys)	Pathogenic	Early infantile epileptic encephalopathy (EIEE)	[188]
CDS	*WDR62*	Deletion/frameshift	NM_001083961.1(WDR62):c.3839_3855del17 (p.Gly1280Alafs)	Pathogenic	Malformations of cortical development	[189]
3′ UTR	*MECP2*	SNV	NM_004992.3(MECP2):c.*2956G > A	Uncertain significance	Rett syndrome	[190]
Promoter	*CRH*	SNV	NC_000008.11:g.66178947G > T	Pathogenic	Familial autosomal dominant nocturnal frontal lobe epilepsy	[191]
Splice site	*ATP6AP2*	SNV	NM_005765.2(ATP6AP2):c.321C > T (p.Asp107=)	Pathogenic	X-linked mental retardation and epilepsy due to inefficient inclusion of exon 4	[192]
Poly(A)	*ARSA*	SNV	NM_000487.5(ARSA):c.*96A > G	Pathogenic	Metachromatic leukodystrophy	[193]
NMD	*SNRPB*	SNV	NM_003091.3(SNRPB):c.-72C > A	Pathogenic	Cerebro-costo-mandibular syndrome	[194]
lncRNA	*ATXN8OS*	Insertion	NR_002717.2(ATXN8OS):n.1103_1105CTG(15_40)	Pathogenic	Spinocerebellar ataxia type 8	[195]

*ACMG* American College of Medical Genetics and Genomics, *CDS* coding sequence, *HGVS* Human Genome Variation Society, *lncRNA* long non-coding RNA, *NMD* nonsense-mediated decay, *SNV* single-nucleotide variant, *UTR* untranslated region

## Classifying genes and other genomic elements

Current gene sets identify under 20,000 protein-coding genes and over 15,000 long non-coding RNAs (lncRNAs) [29, 30]. In this section, for clinicians who might not be familiar with gene structure and function, we present the important elements of different parts of protein-coding genes, and other categories of genomic elements, such as pseudogenes and elements of the non-coding genome such as lncRNAs, and we highlight their potential functionality, illustrated with examples of their roles in disease. We demonstrate the importance of classifying such regions correctly and why incorrect classification could impact the interpretation of sequence variation.

### Important elements of protein coding genes

**Fig. 2:**
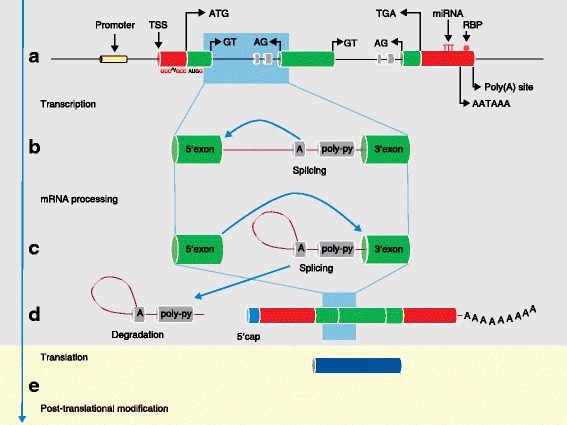
The generic gene model (not to scale). **a** The exons comprise the untranslated regions (UTRs), which are shown in *red* (the 5′ UTR depicted on the *left* and the 3′ UTR depicted on the *right*) and the coding sequence (CDS), which is shown in *green*. Many important regulatory regions lie outside of the exons of a gene. Intronic regulatory regions are shown in *grey*. Promoters are illustrated as *yellow* intergenic regulatory regions, although some genes have internal transcription start sites. The transcription start site (*TSS*) is positioned at the 5′ end of the UTR, where transcription starts. The 5′ UTRs of genes contain regulatory regions. The CDS start codon is the first codon of a messenger RNA (mRNA) from which a ribosome translates. The genomic sequence around the start codon often has the consensus sequence gcc**A**cc|**AUG**|**G** (note that the important bases are highlighted here in bold, whereas the most crucial positions are –3 and +4 from the A of the AUG) [197], although, in very rare cases, a non-AUG start codon is used [198]. The stop codon, of which there are three in eukaryotes—UGA, UAG, UAA—is a nucleotide triplet sequence in an mRNA that gives the signal to terminate translation by binding release factors, causing the ribosome to release the peptide chain [199]. The 3′ untranslated region of genes contains regulatory regions. In particular, the 3′ UTR has binding sites for regulatory proteins such as RNA-binding proteins (*RBP*) and microRNAs (*miRNA*). Promoters are DNA sequences, between 100 and 1000 bp in length, where proteins that help control gene transcription bind to DNA [200]. These proteins can contain one or more DNA-binding domains that attach to a specific DNA sequence located next to the relevant gene [201]. Promoters regulate transcriptional machinery by moving it to the right place in the genome, as well as locating the 5′ end of the gene or an internal transcription start site. Approximately 40% of human genes have promoters situated in regions of elevated cytosine and guanine content, termed CpG islands [202]. A subset of promoters incorporate the variable TATA box sequence motif, which is found between 25 and 30 bp upstream of the TSS and is the position at the 5′ end of the UTR where transcription starts [203]. **b**–**d** Pre-mRNA transcribed from DNA contains both introns and exons. An RNA and protein complex called the spliceosome undertakes the splicing out of introns, leaving the constitutive exons. Intronic and exonic splice enhancers and silencers help direct this procedure, such as the branch point (‘*A*’) and a poly-pyrimidine (*poly-py*) tract. The vast majority of introns have a GT sequence at the 5′ end that the branch point binds to. The intron is then cleaved from the 5′ exon (donor site) and then from the 3′ exon (acceptor site) [204] and a phosphodiester bond joins the exons, whereas the intron is discarded and degraded. During the formation of mature mRNA, the pre-mRNA is cleaved and polyadenylated. Polyadenylation occurs between 10 and 30 bp downstream from a hexamer recognition sequence that is generally AAUAAA, or AUUAAA, although other hexamer signal sequences are known [35] (as depicted in **a**). A specially modified nucleotide at the 5′ end of the mRNA, called the 5′ cap, helps with mRNA stability while it undergoes translation. This capping process occurs in the nucleus and is a vital procedure that creates the mature mRNA. **e** The translation of mRNA into protein by ribosomes occurs in the cytosol. Transfer RNAs (tRNAs), which carry specific amino acids, are read by the ribosome and then bound in a complementary manner to the mRNA. The amino acids are joined together into a polypeptide chain to generate the complete protein sequence for the coding sequence of the transcript. (*Light blue background shading* shows processes that occur in the nucleus. *Light yellow background shading* shows processes that occur in the cytosol, such as the translation of mRNAs into protein by ribosomes)

A eukaryotic gene is typically organized into exons and introns (Fig. *2*), although some genes, for example *SOX3*, which is associated with X-linked mental retardation [31], can have a single exon structure. The functional regions of protein-coding genes are typically designated as the coding sequence (CDS) and the 5′ and 3′ untranslated regions (UTRs) (Fig. *2*).

The 5′ UTR of a transcript contains regulatory regions. For example, some upstream open reading frames (uORFs; which are sequences that begin with an ATG codon and end in a stop codon, meaning that they have the potential to be translated) in the 5′ UTR are translated to produce proteins that could enhance or suppress the function of the main CDS [32]. Experimental techniques such as cap-analysis gene expression (CAGE) [33] are used to identify transcription start sites (TSSs) (Fig. *2*a).

Variants in the CDS are generally the most well studied and understood area of pathogenic sequence variation. For example, approximately 700 pathogenic CDS variants have been reported in the epilepsy-associated gene *SCN1A* [34].

The 3′ UTR of a transcript can contain regions controlling regulatory proteins such as RNA binding proteins (RBPs) and microRNAs (miRNAs) (Fig. *2*a). Interestingly, the 3′ UTR has been linked to overall translation efficiency and stability of the mRNA [35]. The 5′ and 3′ UTRs can also interact with each other to regulate translation through a closed-loop mechanism [36]. Important sequence motifs involved in controlling the expression of a gene include promoters, enhancers and silencers, which are found in exonic, intragenic and intergenic regions (Fig. *2*a).

**Fig. 3:**
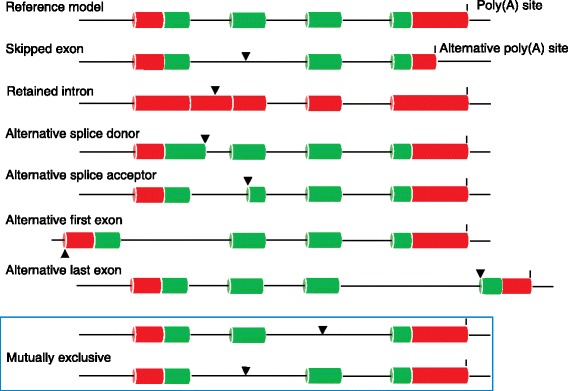
Alternative splicing transcript variants. Different types of alternative splicing can give rise to transcripts that are functionally distinct from a nominal reference model. *Red* represents the untranslated region (UTR) and *green* represents the coding sequence (CDS). The retained intron is illustrated as non-coding as a retained intron is presumed to represent an immature transcript. Some transcripts can contain exons that are mutually exclusive (*boxed*). All the types of alternative exon splicing events shown here can also occur in non-coding genes. There can also be multiple alternative poly(A) features within the gene models, as seen for the skipped-exon transcript

A multi-exonic eukaryotic gene can produce different disease phenotypes through alternative protein isoforms that result from the use of alternative splice site/exon combinations (Fig. *3*) [37]. Canonical splice sites are generally conserved at the 5′ (donor) and 3′ (acceptor) ends of vertebrate introns. The GT–intron–AG configuration is the most common, although other, rarer instances of splice sites are found, such as GC–intron–AG and AT–intron–AC [38].

Although there can be an abundant transcript that is expressed in a particular cell, the same transcript might not dominate elsewhere, and, even if a dominant transcript is identified, the transcript might not be functional [39]. Differential expression can be both tissue- and age-specific [40], can occur in response to different environmental signals [41, 42], and an exon expressed in one tissue might not be relevant to further analysis if it is not expressed in the tissue where a disease phenotype is present. For example, genes expressed in brain generally have longer 3′ UTRs than those in other tissues, and such differences could impact miRNA binding sites and other regulatory regions [43]. Studies have shown that retained introns have an important role in brain gene expression and regulation [44, 45].

Polyadenylation (poly(A)), which involves addition of the poly(A) tail, is important for nuclear export to the cytosol for translation by the ribosome and also helps with mRNA stability (Fig. 2d). Many annotated genes also have more than one poly(A) site, which can be functional in different tissues or different stages of development [42].

After translation, the polypeptide chain produced by the ribosome might need to undergo posttranslational modification, such as folding, cutting or chemical modifications, before it is considered to be a mature protein product (Fig. 2e). Noonan syndrome is believed to result from the disruption of the phosphorylation-mediated auto-inhibitory loop of the Src-homology 2 (SH2) domain during post-translational modification [46].

**Fig. 4:**
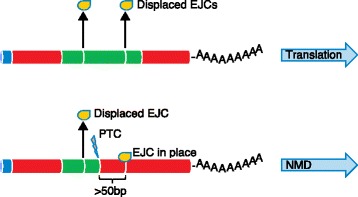
The nonsense-mediated decay (NMD) pathway. Under normal cellular circumstances, exon–exon junction complexes (*EJCs*) that are in place after splicing are removed by the ribosome during the first round of translation. However, when a transcript contains a premature termination codon (*PTC*), perhaps as a result of an single-nucleotide variant (SNV), indel or inclusion of an out-of-frame exon upstream of one or more EJCs, these EJCs remain in place because the ribosome complex disassociates at the premature stop codon and thus cannot remove the downstream EJC. This triggers the NMD pathway, and the transcript is degraded

Transcripts that contain premature stop codons (perhaps as a result of using an alternative splice donor, splice acceptor, or inclusion/exclusion of an alternative exon, which causes a CDS frameshift) are degraded through the nonsense-mediated decay (NMD) cellular surveillance pathway (Fig. 4) [47, 48]. NMD was originally believed to degrade erroneous transcripts, but much evidence has been found to suggest it is also an active regulator of transcription [49, 50]. Several NMD factors have been shown to be important for the regulation of neurological events such as synaptic plasticity and neurogenesis [51–53].

Two other types of cellular surveillance pathways are known to exist: non-stop decay and no-go decay. Non-stop decay is a process that affects transcripts that have poly(A) features but do not have a prior stop codon in the CDS. The translation of such transcripts could produce harmful peptides with a poly-lysine amino acid sequence at the C-terminal end of the peptide—therefore, these transcripts are subject to degradation. Similar to NMD transcripts, either aberrant splicing or SNVs can cause the generation of these transcripts [54]. Finally, no-go decay is triggered by barriers that block ribosome movement on the mRNA [55].

### The functional importance of pseudogenes

**Fig. 5:**
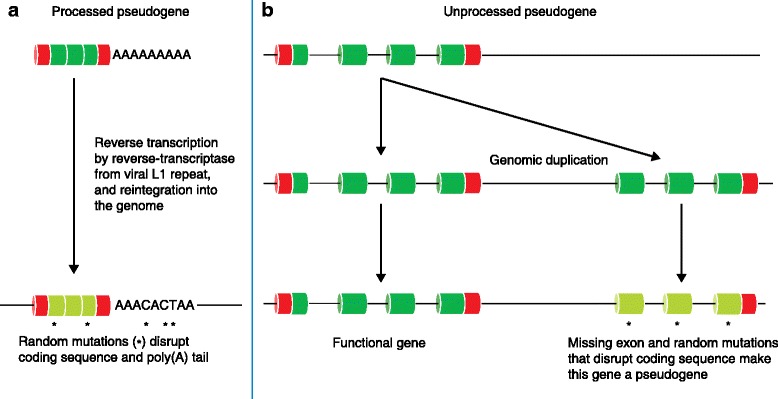
The processes involved in the ‘pseudogenisation’ of genes. **a** Processed pseudogenes are derived from mature mRNA that is reverse-transcribed by the viral L1 repeat enzyme reverse-transcriptase and reintegrated into the genome, and will generally lack introns. Processed pseudogenes are often flanked by direct repeats that might have some function in inserting the pseudogene into the genome and they are often missing sequence compared with their parent. Often they terminate in a series of adenines, which are the remains of the poly(A) tail, which is the site of genomic integration. **b** Unprocessed pseudogenes—the defunct relatives of functional genes—arise from genomic duplication. Such duplications can be complete or partial with respect to the parent gene

Pseudogenes are traditionally regarded as ‘broken’ copies of active genes. Freed of selective pressure, they have typically lost the ability to encode functional proteins through the occurrence of nonsense variations, frameshifts, truncation events, or loss of essential regulatory elements. The majority of pseudogenes fall into one of two categories: processed and unprocessed (Fig. 5, Table 2) [56].

**Table 2:** GENCODE annotation biotypes (2017)

Biotype	Description
Protein coding	Contains an ORF that has strong coding potential
Known coding	100% identical to known RefSeq protein or Swiss-Prot entry
Novel coding	Shares >60% length with known coding sequence from RefSeq, or Swiss-Prot, or has cross-species/family support or domain evidence
Putative coding	Shares <60% length with known coding sequence from RefSeq, or Swiss-Prot, or has an alternative first or last coding exon
Nonsense-mediated decay	If the coding sequence (following the appropriate reference) of a transcript finishes >50 bp from a downstream splice site, then it is tagged as NMD. If the variant does not cover the full reference coding sequence, then it is annotated as NMD if NMD is unavoidable—i.e. no matter what the exon structure of the missing portion is, the transcript will be subject to NMD
Non-stop decay	Transcripts that have poly(A) features (including signal) without a prior stop codon in the CDS—i.e. a non-genomic poly(A) tail attached directly to the CDS without a 3′ UTR; these transcripts are subject to degradation
Retained intron	Alternatively spliced transcript believed to contain intronic sequence relative to other, coding, variants
Processed transcript	Cannot assign an ORF, but is part of a coding locus
lncRNA	Long non-coding RNA—lacks protein-coding potential and is of length >200 bp
Bidirectional promoter	Transcription start sites of the lncRNA model and the protein-coding model are on opposite strands and within 200 bp of one another, or are found in the same CpG island
3-Prime overlapping	Transcription start site and/or published experimental data support independent transcription from the 3′ UTR of a coding gene
Antisense	At least one variant overlaps a protein-coding locus on the opposite strand, or evidence of antisense regulation of a coding gene has been published
lincRNA	Long intergenic ncRNA: does not overlap (neither sense nor antisense) a coding gene
Sense intronic	In an intron of a coding gene; no exonic overlap
Sense overlapping	Contains a coding gene in an intron; no exonic overlap.
Pseudogene	Matches to protein, but ORF disrupted by frameshifts and/or premature stop codons
Processed	Lacks introns and arose from retrotransposition of parent gene mRNA
Unprocessed	Can contain introns and is produced by genomic duplication
Transcribed	Locus-specific transcripts indicate transcription; these can be classified into ‘*processed*’ and ‘*unprocessed*’
Translated	Locus-specific protein mass spectroscopy data suggest translation; the connection is maintained with the pseudogene biotype until the experimental community validates it as a coding gene
Polymorphic	Pseudogene owing to a single-nucleotide variant (SNV), or insertion-deletion variant (indel); but the same gene is translated in other individuals/haplotypes/strains
Unitary	Species-specific unprocessed pseudogene, without a parent gene, that has an active orthologue in another species

Data sourced from GENCODE project [196]

*ncRNA* noncoding RNA, *ORF* open reading frame, *UTR* untranslated region

Processed pseudogenes represent back-integration or retrotransposition of an RNA molecule into the genome sequence, and, although they generally lack introns, they frequently incorporate the remains of the poly(A) tail. Processed pseudogenes are often flanked by direct repeats that might have some function in inserting the pseudogene into the genome, and are often missing sequence compared with their parent gene (Fig. 5) [57]. By contrast, unprocessed pseudogenes are defunct relatives of functional genes that arise through faulty genomic duplication resulting in missing (parts of) exons and/or flanking regulatory regions (Fig. 5).

Computational annotation of pseudogenes tends to suffer from significant false positives/negatives and can cause problems that result from the misalignment of NGS data. Specifically, identification of transcribed pseudogenes and single-exon pseudogenes can be a challenge [58]. Such difficulties were demonstrated where it was found that more than 900 human pseudogenes have evidence of transcription, indicating functional potential [58, 59]. Consequently, the ability to distinguish between pseudogenes and the functional parent gene is essential when predicting the consequence of variants.

MacArthur and colleagues [60] reported that reference sequence and gene annotation errors accounted for 44.9% of candidate loss-of-function (LoF) variants in the NA12878 genome, which belongs to the daughter from a trio of individuals belonging to the CEPH/Utah pedigree whose genomes were sequenced to high depth as part of the HapMap project [61]. The NA12878 genome sequence and transformed cells from the same individual (the GM12878 cell line) are often used as a reference in other projects [62, 63]. After reannotation of protein-coding genes harbouring 884 putative LoF variants, 243 errors in gene models were identified, 47 (19.3%) of which were updated from protein-coding to pseudogene, removing a significant source of false-positive LoF annotation [60].

Transcripts derived from the pseudogene locus *PTENP1* have been shown to regulate the parent *PTEN* locus [64]. Deletion of *PTENP1* has been reported to downregulate *PTEN* expression in breast and colon cancer [64] and melanoma [65], and downregulation of *PTENP1* through methylation of its promoter sequence in clear-cell renal cell carcinoma suppresses cancer progression [66]. Although *PTENP1* has not yet been associated with any neuronal disorders, both *PTEN* and *PTENP1* are expressed in multiple brain tissues [67, 68].

### The non-coding genome

Most of the genome is non-coding, and therefore most variation occurs in non-coding regions. To understand the effect of a sequence variant in such regions, the non-coding elements need to be classified. Non-coding elements consist of *cis*-regulatory elements such as promoters and distal elements (for example, enhancers) [69] and non-coding RNAs (ncRNAs). Large collaborative initiatives, such as ENCODE [63] and RoadMap Epigenomics [70], have been tasked to create comprehensive maps of these regions. The Ensembl regulatory build [71] and Variant Effect Predictor (VEP) [72] are able to determine whether variants fall within such regions, but are not yet able to determine pathogenicity, although tools that do so are beginning to emerge, such as FunSeq [73] and Genomiser [74].

The ncRNAs are generally divided into two groups, small RNAs (sRNAs) and lncRNAs. sRNAs include miRNAs, Piwi-interacting RNAs (piRNAs), short interfering RNAs (siRNAs), small nucleolar RNAs (snoRNAs) and other short RNAs [75]. The sRNAs can be predicted using tools such as Infernal [76] and Rfam [77], which makes the interpretation of sequence variation and consequence easier, especially when compared with the analysis of lncRNAs. However, correctly discriminating functional copies from pseudogenes remains a challenge.

Of particular interest to the study of neurological disease are microRNAs (miRNAs), which are small (approximately 20 nucleotides) ncRNAs that are involved in the regulation of post-transcriptional gene expression [78]. miRNAs can trigger transcript degradation, modify translational efficiency and downregulate gene expression by triggering epigenetic changes (DNA methylation and histone modifications) at the promoter of target genes, and are the best-understood of the ncRNAs. Studies have shown that variants in miRNA binding sites are associated with some neurological diseases, and there is evidence for a role in epilepsy, suggesting that miRNAs might be good candidates for the development of novel molecular approaches for the treatment of patients with epilepsy [79, 80]. For example, miRNA *MIR328* binds to the 3′ UTR of *PAX6* to regulate its expression. However, variation in the miRNA binding site reduces the binding affinity of *MIR328*, which in turn results in an increase in the abundance of *PAX6* transcripts, which is associated with electrophysiological features of Rolandic epilepsy [81]. The EpiMiRNA consortium is investigating the role of miRNAs in the development, treatment and diagnosis of temporal lobe epilepsy [82].

**Fig. 6:**
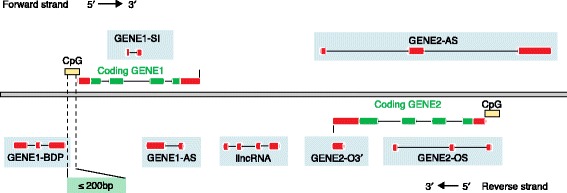
Different classifications of long non-coding RNAs (lncRNAs). The classification of lncRNAs is based on their position with respect to coding genes. lncRNAs are illustrated here with only *red* exons, whereas the coding genes are shown as *red* and *green. AS* antisense, *BDP* bi-directional promoter, *lincRNA* long-intergenic RNA (not overlapping a protein-coding locus on either strand), *OS* overlapping sense, *O3′* overlapping 3′, *SI* sense intronic. Figure adapted from Wright 2014 [84]

The classification of lncRNAs is increasingly used to convey functional information, despite the fact that we know relatively little about the role or mechanism of the vast majority of them (Fig. 6). The term lncRNA was itself established to distinguish longer ncRNAs from the small ncRNAs that were initially separated using an experimental threshold of >200 nucleotides, which remains the simplest definition of a lncRNA [63]. RNA sequencing (RNA-Seq) assays predict that potentially tens, if not hundreds, of thousands of lncRNA transcripts have now been identified [83], which has inevitably led to the naming of many proposed subclasses of lncRNA [84, 85]. Without any international agreement on the classification of lncRNAs, proposed subclasses have been classified based on either length, function, sequence or structural conservation, or association with either protein-coding genes, DNA elements, subcellular location or a particular biological state. They are hard to predict owing to their size, but also because they are expressed at low levels and lack a known tertiary structure, unlike miRNAs. A recent study by Nitsche and colleagues showed that >85% of lncRNAs have conserved splice sites that can be dated back to the divergence of placental mammals [86].

lncRNAs, such as *XIST* [87], have been studied for some time, yet little is known about the function of most. However, they are gaining interest within the scientific and medical community [63] owing to their potential involvement in disease [88, 89]. Experiments in mouse models have demonstrated that dysregulation of certain lncRNAs could be associated with epilepsy [90], and a role in gene regulation is proposed for the vast number of unstudied cases [91], which makes them interesting candidates for new targeted therapies and disease diagnostics [92]. For example, experiments in a knock-in mouse model of Dravet syndrome have shown that the upregulation of the healthy allele of *SCN1A* by targeting a lncRNA improved the seizure phenotype [93].

CNVs also play an important role in human disease and can affect multiple coding genes, resulting in dosage effects, truncation of single genes or novel fusion products between two genes. CNVs have also been shown to be pathogenic in non-coding regions [94]. Talkowski and colleagues [95] observed a CNV causing disruption in the long-intergenic non-coding RNA (lincRNA) *LINC00299* in patients with severe developmental delay, raising the possibility that lincRNAs could play a significant role in developmental disorders. More recently, Turner et al. [96] reported WGS of 208 patients from 53 families with simplex autism and discovered small deletions within non-coding putative regulatory regions of *DSCAM*, implicated in neurocognitive dysfunction in Down syndrome. These CNVs were transmitted from the mother to the male proband.

Repetitive sequences and transposable elements are known to be involved in disease and are believed to make up more than two-thirds of the human genome. They also have a strong association with genomic CNVs [97]. Long interspersed nuclear elements (LINEs) and *Alu* repeats (which are types of retrotransposons) have been associated with increased genomic instability through non-allelic homologous recombination events and can lead to pathogenic duplications and deletions [98]. *Alu–Alu* repeat recombinations within the introns of *ALDH7A1* have been associated with pyroxidine-dependent epilepsy [99]. The ability to accurately detect repetitive sequences is of great importance due to the problems they can cause during the aligning or assembling of sequence reads [100], and the human genome is commonly analysed for repeats using Repbase annotation [101] and computational algorithms, such as the hidden Markov model (HMM)-derived database Dfam [102].

## Genome annotation

The ability to understand the function of a gene and how variation might affect its function is dependent upon understanding its structure, which can be elucidated by genome annotation. Genome annotation in its simplest form proceeds by ab initio gene prediction algorithms that search a genome for putative gene structures [103–105] such as signals associated with transcription, protein-coding potential and splicing [106]. Although these gene-prediction algorithms were used in the early analysis of the human genome [107, 108], they are limited in both accuracy and coverage [29]. The current automated gene-annotation tools, such as Ensembl, provide fast computational annotation of eukaryotic genomes using evidence derived from known mRNA [109], RNA-Seq data [110] and protein sequence databases [111].

Computational annotation systems are essential for providing an overview of gene content in newly sequenced genomes and those with fewer resources assigned to annotation, yet manual annotation is still regarded as the ‘gold standard’ for accurate and comprehensive annotation (Table 3) [112]. As part of the ENCODE project, which was established to investigate all functional elements in the human genome [113], a genome-annotation assessment project was developed to assess the accuracy of computational gene annotation compared with a manually annotated test-set produced by the Human and Vertebrate Analysis and Annotation (HAVANA) team [29]. Although the best computational methods identified ~70% of the manually annotated loci, prediction of alternatively spliced transcript models was significantly less accurate, with the best methods achieving a sensitivity of 40–45%. Conversely, 3.2% of transcripts only predicted by computational methods were experimentally validated.

**Table 3:** Comparison of computationally derived annotation versus manually derived annotation

Annotation procedure	Automatic annotation—for example, Ensembl	Manual annotation—for example, HAVANA
Genome analysis	Very quick	Very slow and labour intensive
Annotation consistency	Consistent	Risk of subjectivity—achieving consistency requires careful training and monitoring
Sequence quality	Flexible; can use unfinished, short-read NGS sequence, shotgun assembly	Best results on high-quality sequence, but can offer great insight into lower-quality assembly
Functional annotation	Limited, lacking comprehensive detail of manual annotation—frequently misassign related sequences—i.e. protein-coding loci and pseudogenes	Extensive use of biotypes, such as coding, pseudogene, lncRNA, NMD, etc.
Complex genomic regions	Limited in ability to represent complex structures and other nonstandard features	Superior representation and resolution of gene families and able to define CDS regions of complicated gene structures
Gene annotation	Many false-positive and false-negative calls at locus level in all gene biotypes	Better coverage of loci and alternatively spliced transcripts
Pseudogenes	Limited	Able to predict pseudogenes and differentiate from genuine coding genes
Poly(A) features	Limited	Annotates poly(A) features
Flexibility	Error prone, forces problems such as non-canonical splicing and can only look at sequences more or less in isolation	Deals with inconsistencies in data, consults literature and other databases, can compare paralogues and orthologues and rapidly integrate new sequencing technologies

*CDS* coding sequence, *HAVANA* Human and Vertebrate Analysis and Annotation, *lncRNA* long non-coding RNA, *NGS* next-generation sequencing, *NMD* nonsense-mediated decay

Only two groups, HAVANA and Reference Sequence (RefSeq) [30], produce genome-wide manual transcript annotation. The HAVANA team is based at the Wellcome Trust Sanger Institute, UK, and provides manual gene and transcript annotation for high-quality, fully finished ‘reference’ genomes, such as that of human [3]. HAVANA manual annotation is supported by computational and wet lab groups who, through their predictions, highlight regions of interest in the genome to be followed up by manual annotation, identify potential features missing from annotation and experimentally validate the annotated transcripts, then provide feedback to computational groups to help improve the analysis pipelines.

The RefSeq collection of transcripts and their associated protein products is manually annotated at the National Center for Biotechnology Information (NCBI) in the USA. Although many RefSeq transcripts are completely manually annotated, a significant proportion are not: for example in NCBI Homo sapiens Annotation Release 106, approximately 45% of transcripts were classified as being computationally annotated [114]. Furthermore, unlike HAVANA transcripts, which are annotated on the genome, RefSeq transcripts are annotated independently of the genome and based upon the mRNA sequence alone, which can lead to difficulty mapping to the genome.

The GENCODE [58] gene set takes advantage of the benefits of both manual annotation from HAVANA and automated annotation from the Ensembl gene build pipeline by combining the two into one dataset. GENCODE describes four primary gene functional categories, or biotypes: protein-coding gene, pseudogene, lncRNA and sRNA. The adoption of further biotypes, at both the gene level and transcript level, has enriched annotation greatly (Table 2). The final gene set is overwhelmingly manually annotated (~100% of all protein-coding loci and ~95% of all transcripts at protein-coding genes are manually annotated). Computational annotation predictions of gene features are provided to give hints to manual annotators and direct attention to unannotated probable gene features, and are also used to quality control (QC) manual annotation to identify and allow correction of both false-positive and false-negative errors.

GENCODE and RefSeq collaborate to identify agreed CDSs in protein-coding genes and to try and reach agreement where there are differences as part of the collaborative Consensus CoDing Sequence (CCDS) project [115, 116]. These CDS models, which do not include 5′ or 3′ UTRs, are frequently used in exome panels alongside the full RefSeq and GENCODE gene sets that form the majority of the target sequences in exome panels.

The GENCODE gene set improves on the CCDS set as it is enriched with additional alternatively spliced transcripts at protein-coding genes as well as pseudogene and lncRNA annotation, and as such is the most detailed gene set [117]. GENCODE is now incorporated into the two most widely used commercial WES kits [118, 119], with fewer variants of potential medical importance missed [120].

**Fig. 7:**
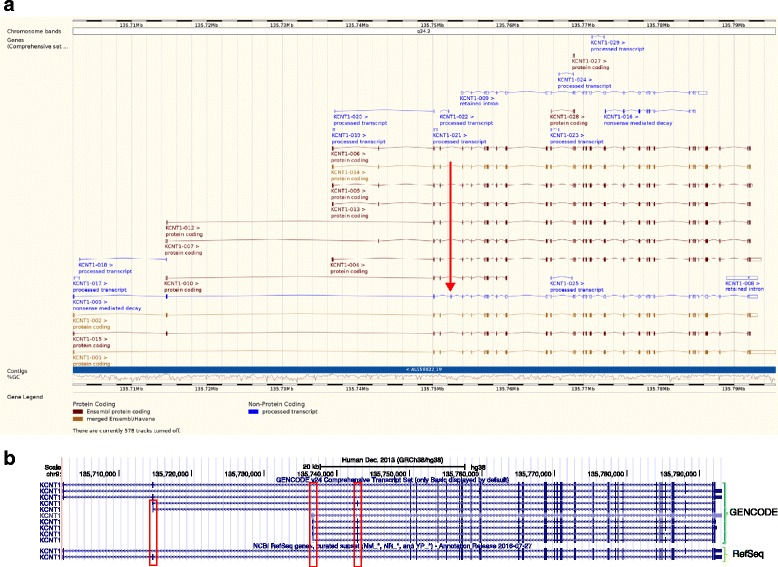
Examples of genome browsers. **a** Screenshot of Ensembl genome browser showing the transcript splicing variants for the gene *KCNT1* encoding a potassium channel subunit. *Gold-coloured* transcripts are those that are found by both manual and computational annotation. *Black* transcripts are those that have been identified only through manual annotation. *Blue* transcripts are annotated without a coding sequence (CDS). For example, the *red arrow* highlights an exon that causes a premature stop codon. This transcript has therefore been identified as being subject to nonsense-mediated decay. **b** Screenshot of the UCSC genome browser also showing *KCNT1*. Comparison of, first, the basic GENCODE gene annotation set (generally full-length coding transcripts based on full-length cDNAs) and, second, RefSeq manually curated genes, which generally have fewer transcripts than GENCODE. The *red boxes* highlight novel transcription start site exons and novel internal exons that are not present in RefSeq

To present genome annotation in a meaningful and useful manner, publicly available, web-based interfaces for viewing annotation have been provided—for example, the Ensembl Genome Browser [71] and the UCSC browser [121] (Fig. 7), both of which display the GENCODE models. The GENCODE genes are updated twice a year, whereas CCDS is updated at least once a year. All transcripts are assigned a unique stable identifier, which only changes if the structure of the transcript changes, making the temporal tracking of sequences easy.

A great deal of functionality is provided by genome browsers, such as: displaying and interrogating genome information by means of a graphical interface, which is integrated with other related biological databases; identifying sequence variation and its predicted consequence using VEP; investigating phenotype information and tissue-specific gene expression; and searching for related sequences in the genome using BLAST. Figure 7 presents by way of example the gene *KCNT1*, which is associated with early infantile epileptic encephalopathies [122] displayed in both the Ensembl and UCSC genome browsers.

## Using comparative genomics to confirm gene functionality

Sequence data from other organisms are essential for interpreting the human genome owing to the functional conservation of important sequences in evolution [123] that can then be identified by their similarity [124]. The zebrafish, for example, has a high genetic and physiological homology to human, with approximately 70% of human genes having at least one zebrafish orthologue. This means that the zebrafish model can provide independent verification of a gene being involved in human disease. Zebrafish also develop very quickly and are transparent, and so the fate, role and life cycle of individual cells can be followed easily in the developing organism. This makes the zebrafish a highly popular vertebrate model organism with which to study complex brain disorders [125, 126], and it has been essential for modelling disease in the DDD study [127].

Likewise, owing to a combination of experimental accessibility and ethical concerns, the mouse is often used as a proxy with which to study human disease [128, 129], and this justified the production of a high-quality, finished, reference mouse genome sequence, similar to that of the human sequence [130]. Murine behavioural traits, tissues, physiology and organ systems are all extremely similar to those of human [131], and their genomes are similar too, with 281 homologous blocks of at least 1 Mb [132] and over 16,000 mouse protein-coding genes with a one-to-one orthology to human [133]. The large number of knockout mouse models available can be used to study many neurological diseases in patients [128], such as the Q54 transgenic mouse used to study *Scn2A* seizure disorders [134]. Recent studies in rodent models of epilepsy have identified changes in miRNA levels in neural tissues after seizures, which suggests that they could be key regulatory mechanisms and therapeutic targets in epilepsy [135]. It is therefore important that high-quality annotation for these model organisms is maintained, so that genes and transcripts can be compared across these organisms consistently [136]. With the advent of CRISPR–Cas9 technology, it is now possible to engineer specific changes into model organism genomes to assess the effects of such changes on gene function [137].

Nevertheless, model organism genomes and human genomes differ. For example, the laboratory mouse is highly inbred, whereas the human population is much more heterogeneous [138]. Furthermore, many environmental and behavioral components are known to affect disease in certain mouse strains, which are factors that are not clearly understood in human disease [139]. Although comparative genomics helps to build good gene models in the human genome and understand gene function and disease, basing predictions in clinical practice upon animal models alone might lead to misdiagnosis.

## New techniques to improve functional annotation of genomic variants

NGS technologies facilitate improvements in gene annotation that have the potential to improve the functional annotation and interpretation of genomic variants. The combination of both long and short NGS reads [140] will change the scope of annotation. While short-read RNA-Seq assays may be able to produce hundreds of millions of reads and quantify gene expression, they are generally unable to represent full-length transcripts, which makes the assembly of such transcripts incredibly difficult [141]. However, the greater read lengths produced by new sequencing technologies such as PacBio and synthetic long-read RNA-Seq (SLR-Seq), which uses Illumina short-read sequencing on single molecules of mRNA, have the potential to produce sequence for complete transcripts in a single read. In addition, utilizing longer-read technologies such as that from PacBio has already been shown to improve resolution of regions of the genome with SVs [142], and emerging technologies, such as 10X genomics [143], promise further improvements. This is especially important because WES is unable to represent structural variation reliably. The importance of representing such regions through WGS has been demonstrated by numerous neurological diseases associated with SVs, including cases of severe intellectual disability [144]. Other examples of SV-induced neurological disease include Charcot–Marie–Tooth disease, which is most commonly caused by gene-dosage effects as a result of a duplication on the short arm of chromosome 17 [145], although other causes are known [146]; Smith–Magenis syndrome, caused by copy-number variants on chromosome 17p12 and 17p11.2 [147]; and Williams–Beuren syndrome, caused by a hemizygous microdeletion involving up to 28 genes on chromosome 7q11.23 [148].

Together, NGS data will also lead to the discovery of new exons and splice sites that both extend and truncate exons in a greater diversity of tissues and cell types. Whether the variants identified that are associated with novel exons or splice sites belong to protein-coding transcripts, or potential regulatory transcripts, or are transcripts likely to be targets of the NMD pathway, such technologies will permit better functional annotation of these overlapping variants. An example is the re-annotation of variants that were previously called intronic as exonic sequences. Similarly, a previously described synonymous substitution, or benign non-synonymous substitution, could affect core splice-site bases of a novel splice junction. RNA-Seq assays are able to discern expression of individual exons, allowing prioritisation of variants expressed in appropriate tissues for a disease. In the future, clinical investigation could target the genome in conjunction with the transcriptome—for example, using patient tissue as the basis for RNA-Seq assays—to identify regions where genes are expressed irregularly.

Transcriptomics datasets, such as CAGE [33], RAMPAGE [149] and polyA-seq [150], aid the accurate identification of the 5′ (for the two former) and 3′ (for the latter) ends of transcripts. This knowledge allows researchers to better annotate the functionality of a biotype, specifically enabling the addition of CDS where this was not previously possible, and enriching the functional annotation of overlapping variants. Furthermore, knowledge of termini allows the confident annotation of 5′ and 3′ UTRs that could harbor important regulatory sequences such as uORFs and miRNA target sites.

Other datasets, such as mass spectrometry (MS) [151] and ribosome profiling (RP, or Riboseq) [152], indicate translation, either by directly identifying proteins (MS) or by identifying translation on the basis of ribosomal binding to mRNA transcripts (RP), which aids the accurate identification of the presence and extent of expression of the CDS. Combining these datasets with cross-species conservation of protein coding potential found by PhyloCSF [153] allows annotators to identify previously unannotated protein-coding loci and confirm lncRNAs as lacking in protein-coding potential.

With the increasing importance of epigenetics and its role in neurological disorders [154], such as epilepsy [155], several companies are making detection of these features a priority—for example, detecting methylated nucleotides directly, as part of their sequencing reaction [156]. Other well-described genetic marks are the DNase hypersensitivity sites that are often found in regions of active transcription [63]. However, before these marks are considered in the process of annotation, we will require better experimental datasets that validate them. To put such marks into context and aid validation, gene annotation must be as accurate and comprehensive as possible so that potential *cis* (local) and *trans* (distant) interactions can be identified. Regulatory regions such as enhancers are features that can be described as part of the extended gene and represent the next frontier for gene annotation using data such as Capture Hi-C [157] and ChIA-PET [158] to identify physical connections between regulatory regions affected by variation and the genes they regulate, which can often be located a great distance away. This could mean that variants that were previously considered to be benign could in future be reclassified as pathogenic. For example, variants in evolutionarily conserved transcription factor binding sites are believed to have a role in narcolepsy [159].

Computational and manual genome-annotation methods that have been described have relied almost exclusively on traditional transcriptional evidence to build or extend models of genes and their transcripts. While the number of sequences in public databases continues to increase, genes expressed at very low levels, or with restricted expression profiles (such as many non-coding loci), are likely to remain either under-represented or incomplete when relying on such evidence [160, 161].

New technologies and software will help assess the complexity of loci much more thoroughly through the investigation of alternative splicing/translation start sites/poly(A) sites [162], alternative open reading frames, and so on. They will also allow the revisiting of the human genome—for example, to investigate evolutionarily conserved regions and regulatory features for functionality and to identify new non-coding loci structures as well as new coding transcripts.

## Conclusions

We have reviewed how important regions of the genome that harbor pathogenic sequence variation can lie outside the CDS of genes. We have discussed how researchers can better understand why an incorrect interpretation of a pathogenic variant could arise. Such reasons can range from the human reference genome being incomplete, not all exons being represented in public databases, to incorrect annotation of transcripts/exons owing to their expression in a different tissue or at a different developmental stage to the disease phenotype. Table 4 gives a summary of such examples. As such, considerable efforts continue to be made to increase the catalogue of new genes involved in diseases, such as neurological disease [127]. However, even well-studied genes should be revisited iteratively to identify novel features that previous technology could not detect. For example, a recent publication by Djemie and colleagues [163] revisited patients who had presented with Dravet syndrome, typically associated with *SCN1A* variants, but had been *SCN1A* variant-negative after clinical sequencing. By re-testing with NGS, it was possible to identify 28 variants that were overlooked with Sanger sequencing. Around 66% of the reported false-negative results were attributed to human error, whereas many of the others were a result of poor base-calling software [164].

**Table 4:** Important areas to consider for genome annotation

Genome assembly is not complete	Human assembly is still not complete and still being refined The current assembly is GRCh38, which still contains fragmented genes, and gene duplications are incorrectly represented, yet most analysis is still performed on GRCh37
Transcriptome is still incomplete	Some exons are still not represented in the human genome owing to low expression or temporal expression in tissue that has not yet been interrogated WES kits will not contain all exons WGS-negative cases should be iteratively re-analysed as new transcriptional features are revealed
Reference annotation datasets can be missing key features	Automatic annotation is fast but not as accurate as manual annotation CCDS—missing UTRs LRG—single, usually canonical, transcript—potential for missing exons; choice of transcript is arbitrary RefSeq—based on transcriptome, potential for missing exons and problems with inconsistent mapping to reference assembly Annotation does not necessarily determine which transcripts are the most likely to be functional, and the longest one might not be the major one
Non-coding genome	Long-range gene interactions are poorly understood; methods such as Capture Hi-C will provide insights into such epigenetics Previously ignored transcript biotypes such as NMD and retained intron are now known to have important regulatory roles in disease Non-coding RNAs have an important role in disease, yet they are hard to predict and their function remains largely unknown.
Biotype associations	A biotype conflict in annotation datasets will cause incorrect variant calls—for example, lncRNA variant compared with coding gene, coding gene compared with pseudogene
Transcript expression profile	Is transcript expressed in correct tissue for disease phenotype? Is transcript expressed at the right developmental time for disease phenotype?

*CCDS* Collaborative Consensus Coding Sequence project, *lncRNA* long non-coding RNA, *LRG* Locus Reference Genomic project, *NMD* nonsense-mediated decay, *WES* whole-exome sequencing, *WGS* whole-genome sequencing

It is important to remember that the full human transcriptome has yet to be annotated across all tissues of the human genome. Clearly, while gene panels and whole-exome sequences are a great start to getting a diagnosis, they are not perfect as they are snapshots of sequence at a particular point in time, meaning that pathogenic sequence variants that lie in yet-to-be-annotated exons will not be detected. This emphasizes the power of whole-genome sequences as, unlike exomes, they can be re-analysed again at any point in the future as new gene structures are found [165]. To identify such features, it will be important to update the annotation of disease genes using the most relevant experimental methods and tissue to help identify transcripts that might be expressed at low levels or only at certain developmental stages.

Similarly, improvements in the understanding and annotation of gene structures can lead to reclassification of variants as less pathogenic than previously believed, with implications for treatment strategies. For example, de la Hoya and colleagues demonstrated that improvements to understanding of native alternative splicing events in the breast cancer susceptibility gene *BRCA1* show that the risk of developing cancer is unlikely to be increased for carriers of truncating variants in exons 9 and 10, or indeed other alleles that retain 20–30% tumour-suppressor function, even where such variants had been previously characterized as pathogenic [166].

Accordingly, it is essential to consider multiple transcripts for pathogenic variant discovery, unlike the standard clinical approach of only considering a ‘canonical’ transcript, invariably based on the longest CDS but not necessarily on any expression values [167]. Such situations could result in ambiguous HGVS nomenclature when transcript IDs are not specified, and, as a result, important variants might be missed if variant analysis is only performed against the canonical transcript. For example, a variant can be classified as intronic based on the canonical transcript but could be exonic when based upon an alternatively spliced transcript. Such technical challenges illustrate the difficulties for clinicians when dealing with clinical reports containing details of identified variants (for example, HGVS identifiers) and attempting to map them accurately to function and allow variant interpretation.

**Fig. 8:**
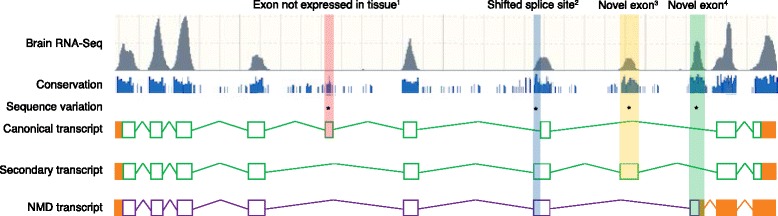
The importance of multiple alternative transcripts for variant interpretation. This hypothetical example of gene ‘*AGENE*’ expressed in brain highlights how the same variant could have different outcomes in different transcripts. We illustrate this further using hypothetical HGVS nomenclature. Note that when there are multiple transcripts for a gene, this can have an effect on amino acid numbering of variants as different transcripts can have different exon combinations, meaning that the same exon in two different transcripts can have a different translation and can also result in different lengths for the amino acid sequence. Note too that the untranslated region is represented by *orange boxes. Green boxes* represent the coding sequence (CDS), whereas *purple boxes* represent the CDS of the nonsense-mediated decay (*NMD*) transcript. *Lines* that join exons represent introns. *Asterisks* indicate the positions of the following hypothetical variants. (1) NM_000000001.99(AGENE):c.2041C > T (p.Arg681Ter). This variant might not be of interest to the clinician as it lies in an exon that is not expressed in brain. (2) NM_000000002.99(AGENE):c.4002 + 2451G > C. The Human Genome Variation Society (HGVS) suggests that this variant is intronic, yet, by looking across other transcripts, it is clear that the variant falls in an extended coding exon that is expressed in brain. (3) NC_000000003.99:g.66178947G > T. This variant is intronic to the canonical transcript, but falls in a well-conserved exon that is expressed in brain. (4) ENSP0000000004.1(AGENE):p.Gly276Ala. This variant falls in an exon that induces NMD. The exon is well conserved and expressed in the brain, making it potentially relevant to the clinician. Generally, NMD transcripts have been considered to be non-coding and excluded from sequence analysis. However, such exons are now known to have an important role in gene regulation. For example, Lynch and colleagues [194] reported that variation in the highly conserved exon in *SNRPB* that induces NMD can result in severe developmental disorders

A solution to this problem would be to identify all the high-confidence transcripts and call variants against these transcripts, highlighting variants that might have severe effects against one or more such transcripts. To improve sensitivity, these findings could be weighted by transcript expression level in the disease-relevant tissue(s) (Fig. 8). To improve sensitivity even further, RNA-Seq assays from different developmental stages could be interrogated to see whether exons are expressed at the correct developmental stage as that of the disease phenotype [63].

Also of interest and concern is where genes thought to be implicated in a specific disease are now thought to have insufficient evidence for their role in disease. For example, the following genes were previously thought to be associated with epilepsy: *EFHC1* [168], *SCN9A*, *CLCN2*, *GABRD*, *SRPX2* and *CACNA1H* [169]. The Epilepsy Genetics Initiative (EGI) attempts to address such problems by iteratively re-analysing WES and WGS of epilepsy cases every 6 months.

The overwhelming amount of sequence variation that is generated by WES and WGS means that many variants produced will have no role in disease. Therefore, the use of databases that contain sequence variants from global sequencing projects, such as ExAC [170] and the 1000 Genomes Project [171] can help filter out common variants to help identify rare variants [60, 172]. Such databases can be used to identify those genes that are intolerant of any variation in their sequence, and, when variants in such genes are identified in patients, this could be an indicator of pathogenic sequence variation [173]. Other variant databases, such as The Human Gene Mutation Database (HGMD) [174] and ClinVar [175], provide information on inherited disease variants and on relationships between variants and phenotype. Genomic interpretation companies are now providing increasingly quick pathogenic variant interpretation turnaround times [176–179]. However, the value of such interpretation will only be as good as the gene annotation that is used for genome analysis and interpretation, demonstrating the need for continual updating and improvement of current gene sets.

Genome annotation is also increasingly seen as essential for the development of pharmacological interventions, such as drug design. Typically, drug design targets the main transcript of a gene (the choice of such a transcript is not necessarily informed by biological data, but is generally based upon the longest transcript), yet, as mentioned previously, it is now understood that certain transcripts can be expressed in different tissues, or at certain developmental times [180]. For example, the onconeural antigen Nova-1 is a neuron-specific RNA-binding protein, and its activity is inhibited by paraneoplastic antibodies. It is encoded by *NOVA1*, which is only expressed in neurons [181]. The alternative splicing of exon 5 of the epilepsy-associated gene *SCN1A* generates isoforms of the voltage-gated sodium channel that differ in their sensitivity to the anti-epileptic medications phenytoin and lamotrigine [180]. Finally, isoform switching in the mouse gene *Dnm1* (encoding dynamin-1), as a result of alternative splicing of exon 10 during embryonic to postnatal development, causes epilepsy [182].

With new drugs having a high failure rate and associated financial implications [183–185], it is not unreasonable to suggest that identifying tissue-specific exons and transcripts through annotation has the potential to reduce such failure rates significantly. New methods of generating genomic data must therefore be adopted continually and interrogated by annotators to facilitate the translation of genomic techniques into the clinic in the form of genomic medicines.

Such advances will begin to address some of the controversies and challenges for clinicians that the fast advances in genomics bring. They will help to understand why current technology can fail to identify the pathogenic basis of a patient’s disorder, or, more worryingly, why it can produce an incorrect result where the wrong variant is labelled as causative. This understanding will help clinicians to explain the advantages and limitations of genomics to families and healthcare professionals when caring for patients. The implication is that it will empower them to request reanalysis of unsolved cases as newer technology improves the annotation of gene structure and function. It will also encourage clinicians to request referral for disease modification when therapy becomes available for a clinical disease caused by specific genomic alterations.

## Abbreviations

ACMG: American College of Medical Genetics and Genomics
CAGE: Cap-analysis gene expression
CCDS: Consensus coding sequence
CDS: Coding sequence
CNV: Copy-number variant
DDD: Deciphering Developmental Disorders
HAVANA: Human and Vertebrate Analysis and Annotation
HGP: Human Genome Project
HGVS: Human Genome Variation Society
indel: Insertion and deletion
lincRNA: Long-intergenic non-coding RNA
lncRNA: Long non-coding RNA
LoF: Loss-of-function
miRNA: MicroRNA
NCBI: National Center for Biotechnology Information
ncRNA: Non-coding RNA
NGS: Next-generation sequencing
NMD: Nonsense-mediated decay
ORF: Open reading frame
PacBio: Pacific Biosciences
RefSeq: Reference Sequence
RNA-Seq: RNA sequencing
sRNA: Small RNA
TSS: Transcription start site
UTR: Untranslated region
VEP: Variant effect predictor
WES: Whole-exome sequencing
WGS: Whole-genome sequencing
